# Evaluation of Different Autologous Platelet Concentrate Biomaterials: Morphological and Biological Comparisons and Considerations

**DOI:** 10.3390/ma13102282

**Published:** 2020-05-15

**Authors:** Enrico Marchetti, Leonardo Mancini, Sara Bernardi, Serena Bianchi, Loredana Cristiano, Diana Torge, Giuseppe Marzo, Guido Macchiarelli

**Affiliations:** 1 Department of Life, Health and Environmental Sciences, University of L’Aquila, 67100 L’Aquila, Italy; enrico.marchetti@univaq.it (E.M.); leonardo.mancini@student.univaq.it (L.M.); serena.bianchi@univaq.it (S.B.); loredana.cristiano@univaq.it (L.C.); diana.torge@graduate.univaq.it (D.T.); giuseppe.marzo@univaq.it (G.M.); guido.macchiarelli@univaq.it (G.M.); 2 Microscopy Center, University of L’Aquila, 67100 L’Aquila, Italy

**Keywords:** platelet-derived biomaterials, cell culture, biocompatibility

## Abstract

The field of regeneration interventions in oral and maxillofacial surgeries still represents a challenge for researchers and clinicians. Understanding the biological and morphological behaviour of human cells towards the materials used for the regeneration surgeries is key to successfully choosing and applying the appropriate biomaterials for specific clinical situations. The aim of the study was the biological and morphological evaluation of autologous platelet concentrate materials obtained with different protocols, in culture with human periodontal ligament fibroblasts (HPLF). The study design included the evaluation of Leukocyte-Platelet-Rich-Fibrin (L-PRF), Concentrated Growth Factors (CGF) and autologous platelet gel (APG) in contact with the HPLF cell line after 24 h, 72 h and 7 days of in vitro culture. Cell proliferation and, therefore, viability were evaluated with XTT assays. The morphological response of the cells was evaluated by light microscopy, scanning electron microscopy and confocal microscopy. The XTT assay showed an interesting response in the growth curve. In particular, the material that gave the best results was the CGF. The morphological data supported the XTT assay, showing the best results for the CGF and L-PRF. In conclusion, all the platelet-derived materials stimulated the onset of the growth of the HPLF cell line, making them promising options for periodontal regeneration interventions.

## 1. Introduction

In recent years, there has been a growing interest in the development of biomaterials for regenerative purposes [1,2] associated with various techniques such as sinus augmentation, ridge preservation and periodontally regenerative procedures [3]. In particular, autologous platelet concentrates (APCs) are often used as biomaterials for the treatment of many intraoral clinical conditions, including periodontal defects [4]. Indeed, the autologous growth factors and the fibrin fibres in the autologous platelet concentrates can be used in surgical wounds to promote faster local healing [5,6]. 

The fibrin is usually a three-dimensional matrix, containing platelets, glycanic chains, cytokines and structural glycoproteins. This structure behaves as a network that appears suitable for the growth of human periosteal cells [7]. It can be used alone, as a form of natural “scaffold”, or together with other graft materials [8]. Several generations of APC have been produced in the last few years, such as platelet-rich plasma (PRP) and plasma rich in growth factors (PRGF), also known as autologous platelet gel (APG) [9]. The need for a second APC generation emerged due to several factors limiting the use and the versatility of PRP and PRGF [7]. Their preparation requires the additional use of bovine thrombin or CaCl_2_ and coagulation factors. Furthermore, the solution must be centrifugated in two separate stages to increase the platelet concentration without the incorporation of leukocytes (sometimes requiring 1 h) [7]. Lastly, the clinical potential for bone regeneration with PRP or APG is limited since they show a very short release profile for growth factors [10,11,12,13] and result in a weak fibrin network [7]. 

Platelet-Rich-Fibrin (PRF) differs from its predecessors according to several parameters; the protocol for its preparation is relatively simple, without blood biochemical manipulation or the need to add anticoagulants. Therefore, the products are easy to use, with a low probability of preparation mistakes [14].

Essentially, PRF is an autologous matrix containing a large amount of leukocyte cytokines and platelets [1,2,7,15,16], which interact with the fibrin clot to form a haemostatic plug and to slowly release growth factors in order to stimulate wound healing. For these reasons, biological materials of this kind have been widely used in medical procedures, such as facial plastic surgery [4,17], skin ulcers [5,18], sinus-lift procedures [6,19], multiple gingival recession cases [7,8,20,21] and periodontal surgery, as well as in intrabony defect [9,22,23] and class II furcation defect treatment [10,24].

In the literature, various types of PRF preparation, based on different relative centrifugation forces (RCFs) have been proposed. The most common is the Leukocyte-PRF or L-PRF by Choukroun et al. [6]. However, it was demonstrated that an increase in the speed of rotation is associated with a higher weight of the pushed-away corpuscles. Therefore, the use of lower centrifugation leads to a greater retention of leukocytes and cytokines and to their better distribution within the fibrin clot. Moreover, a reduction in the RCF may lead to an improvement in the number of leukocytes. Hence, the significant slow and persistent release of key growth factors for ≥1 week and ≤28 days has well-known additive effects on the healing process [18]. Due to the introduction of new protocols and new concepts of spin speed in literature, other types of APC, similar to the original PRF, have been proposed with different characteristics, such as Concentrated Growth Factors (CGF); CGF are a fibrin matrix rich in growth factors prepared with a different speed protocol proposed by Sacco [4]. The speed protocol includes acceleration for 30 s, 2700 rpm for 2 min, 2400 rpm for 4 min, 2700 rpm for 4 min, 3000 rpm for 3 min, deceleration for 36 s, then stopping. 

The different speed of centrifugation results in the production of a high-density matrix, rich in growth factors, as previously reported in immuno-histochemical studies present in the literature [11,25]. The advantages of using CGF in regenerative procedures is the slow release of growth factors, improving the healing process. The tetramolecular fibrin structure, formed during the spin phase, lead cells to migrate, facilitating the replication process.

PRF and second-generation APCs showed a dense fibrin cloth that can reduce the numbers of platelets and, consequently, the number of growth factors trapped in the cloth. Conversely, the data for the PRP and first-generation APCs showed an inexistent fibrin structure at the expense of platelets and growth factors such as APG.

APG is an autologous platelet gel generated with a speed of 480 RCF for 4 min and a phase critical for blood separation including clot activation; it is a matrix that is not simple to manage and that needs 10 to 15 min before being usable [12,26].

The principal characteristics and our considerations according to the preparation protocols are shown in Table 1.

The aim of this study was to evaluate the bio-morphological behavior of human periodontal ligament fibroblasts in response to alternative types of fibrin matrix, L-PRF, CGF and APG and their efficiencies, if any, in regenerative procedures.

## 2. Materials and Methods

### 2.1. Sampling Procedures and APC Preparation

Blood samples were obtained from a healthy, 25-year-old, non-smoking Italian man, after obtaining his written informed consent, in accordance with the Helsinki Declaration on the Ethical Principles for Medical Research Involving Human Subjects.

#### 2.1.1. APC Preparation—L-PRF

Twenty-seven millilitres (ml) of venous blood was collected in one dry glass tubes (9 mL in each) (Blood collecting tubes^®^, Process, Nice, France) without any anticoagulant. According to the standard Choukroun’s protocol [7], tubes were immediately centrifuged at 2700 rpm (approximately 400× *g*) for 12 min with a dedicate centrifuge (Intra-lock International, Birmingham, AL, USA). A fibrin dense clot was then obtained in the middle of the tube, between the red cells at the bottom and the liquid serum called platelet-poor plasma at the top.

#### 2.1.2. APC Preparation—CGF

The protocol to obtain CGF followed Sacco’s protocol [28] using a dedicated centrifuge (CGF, Silfradent, Santa Sofia, FC, Italy). Briefly, the protocol of the centrifugation included acceleration for 30 s, 2700 rpm × 2 min, 2400 rpm × 4 min, 2700 rpm × 4 min, 3000 rpm for 3 min, deceleration for 36 s and then stopping.

#### 2.1.3. APC Preparation—APG

The APG preparation included the collection of approximately 9 mL of blood into collection tubes. The sample was shaken to mix the anticoagulant with the blood. The tubes, containing sodium citrate 3.8%, were then centrifuged at 480 RCF for 4 min with a dedicated centrifuge (GF One, UBGEN, Padova, Italy). After the centrifugation, the blood sample was divided into different parts: the red-coloured fraction containing the red blood cells, the layer rich in platelets, the whitish layer containing white blood cells and the autologous platelet gel (APG) consisting of the autologous fibrinogen.

According to the protocol, the layer rich in platelets was aspirated and the APG was mixed with 10U of CaCl_2_, to transform the liquid phase to gel phase. The total preparation time from venipuncture to injection was approximately 10 min, and 1 mL of APG was produced per tube.

### 2.2. Cell Culture

The human periodontal ligament fibroblast (HPLF) cell line from ScienCell research Laboratories was cultured according to the manufacturer’s instructions. Briefly, the initial vial containing 5 × 10^5^ cells in 1 mL of volume was cultured in three plastic culture dishes in Fibroblast Medium and incubated under standard cell culture conditions (37 °C in 5% CO_2_). One bottle of Fibroblast Medium is composed of the following: 500 mL of basal medium, 10 mL of fetal bovine serum (FBS), 5 mL of fibroblast growth supplement and 5 mL of penicillin/streptomycin solution (10,000 IU/mL of Penicillin, 10 μg/mL Streptomycin). When the cells reached sub-confluence, they were detached using 0.05% trypsin, and subcultured at a density of 110 cells/mm^2^. The cells were used at subculture passages 7 or 8 for all experimental assays.

### 2.3. Cell Proliferation Assays and Statistical Analysis

The cells were grown in 96 well plates under standard cell culture conditions. Two micrograms of the tested material were plated into the 96 well microplates. After 24 h, 72 h and 7 days, the XTT Assay (Cayman Chemical, Ann Arbor, MI, USA) was used to assess the proliferation activity of the cells, and the microplates were read at an absorbance wavelength of 450 nm. The proliferation of the negative control cultures was set at 100%. The XTT tests were performed with three technical replicates in three independent experiments. The mean density of the test groups was divided by that of the control group and expressed as a percentage of the control value.

The grouped raw data were analysed employing two-way ANOVA, using GraphPad Prism 8.4.2 (GraphPad Software, San Diego, CA, USA), considering a *p*-value < 0.05 significant.

### 2.4. Morphological Analysis

The qualitative evaluation of cells was performed by light microscopy (LM), to have a first-sight overview of the culture, then scanning electron microscopy (SEM) and confocal laser scanning microscopy (CLSM), to observe the eventual morphological changes.

#### 2.4.1. Morphological Analysis—LM

Cells were plated in 30 mm diameter plastic culture dishes with the tested and the control material and were incubated under cell culture conditions. After 24 h, 72 h and 7 days from the seeding, three dishes containing the tested material and other material were fixed using a 10% solution of formaldehyde and processed for light microscopy using Azan Mallory staining (Bio-Optica SpA, Milan, Italy), following the instructions of the manufacturer. The Azan Mallory stain is used to process connective tissues and therefore indicated to stain fibroblasts. The observation included the use of a light microscope (Zeiss, AxioImager A2, Jena, Germany) and the use of a Leica DFC 320 camera to capture the images.

#### 2.4.2. Morphological Analysis—SEM

After 24 h, 72 h and 7 days from the seeding of the three dishes, containing cover glasses on which the cell solutions containing the tested material or the test control were seeded, the cells were fixed using a 2% solution of glutaraldehyde and were processed for the SEM, as described previously [29,30,31]. Briefly, after the post fixation in 1% osmium for 1 h, the samples were dehydrated in ascending concentration ethanol solutions of 70%, 80%, 90% and three times 100% for 10 min each. Afterwards, the samples were air-dried by the evaporation of hexamethyldisilane (HDMS). Subsequently, the samples were immersed for 3 min in 100% HDMS (Sigma-Aldrich srl, Milan, Italy). The samples were then transferred into a desiccator for 25 min to prevent water contamination. The samples were mounted on metal stubs, gold stained, and then observed by SEM (GEMINI_SEM, Zeiss, Germany) at different magnifications using secondary electrons and InLens probes.

#### 2.4.3. Morphological Analysis—CLSM

Cells grown on a coverslip in the presence of the considered APCs for 24 h, 72h and 7 days were fixed with 4% paraformaldehyde (Bio-Optica SpA, Milan, Italy) in PBS for 10 min, permeabilised with 0.1% Triton-X-100 for 5 min and incubated with a blocking solution (PBS containing 3% BSA), for 10 min at room temperature (RT). The cells were then incubated with the mouse monoclonal anti-CD90 (Thy-1)/fibroblast primary antibody [32] diluted 1:200 in blocking solution (Chemicon International Inc. Temecula, CA, USA), for 1 h at RT, then incubated with the donkey anti-mouse IgG Alexa Fluor 488 conjugated secondary antibody (1:2000, Immunological Science Rome, Roma, Italy) for 30 min at RT.

For actin staining, cells were incubated with Phalloidin Alexa Fluor 546 diluted 1:100 in the blocking solution (Immunological Science Rome, Roma, Italy), for 30 min, at RT.

Finally, the coverslips were mounted with Vectashield Mounting Medium with 4′,6-diamidino-2-phenylindole (DAPI) (Vector Laboratories, Burlingame, CA, USA) and examined with a Leica TCS SP5 confocal microscope (Mannheim, Germany). Controls were performed by omitting the primary antibody.

## 3. Results

The combined analyses of XTT and the morphological observations allowed a full and integrated overview of the performance of the examined APCs in contact with the HPLF to be obtained.

### 3.1. Cell Proliferation Assays and Statistical Analysis

**Figure 1:**
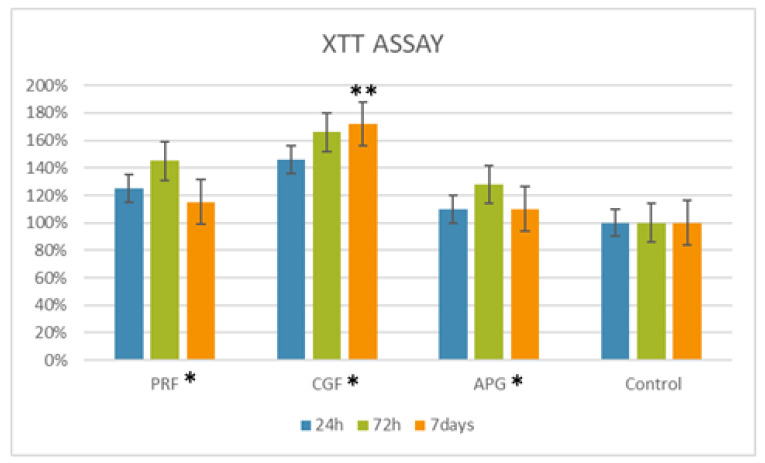
Graphical representation of the growth curve of the human periodontal ligament fibroblast (HPLF) cells in contact with the APCs at different times. For 24 h, the highest peak is registered for the PRF, followed by the CGF and APG. At 72 h and at 7 days, the material with best performance is the CGF, followed by the PRF and APG. The two-way ANOVA shows how the biomaterial proliferation values were statistically significant (*) with a *p*-value < 0.05 compared to the control. Among the materials, the Dunnett’s multiple comparison test showed how the mean of the proliferation with CGF vs. control was statistically significant at 7 days (**).

The proliferation data showed that the cells treated with L-PRF and CGF increased their proliferation at all the considered times with respect to control with 100% viability (Figure 1). In particular, the CGF condition showed a better performance at 24 and 72 h with values of 146% and 166%, respectively, followed by the L-PRF, which showed proliferation values of 125% and 145% at 24 and 72 h, respectively. The APG gave a low level of proliferation with a value of 128% during the first 72 h. The highest HPLF cell proliferation was observed with CGF at 7 days.

As displayed in Figure 1, the two-way ANOVA analysis showed a statistical difference for the “biomaterials” parameter, and the subsequent multiple comparison tests displayed a statistically significant difference for the CGF at 7 days among the considered materials at the examined times (statistical tables, Tables S1 and S2 are available in Supplementary Materials).

### 3.2. Morphological Analysis—LM

**Figure 2:**
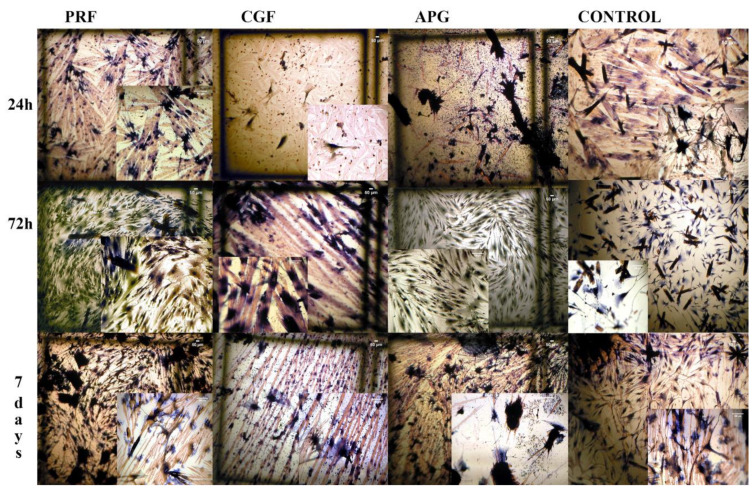
Light microscopy (LM) images of HPLF cells with the examined materials and the controls at the different examined times. The brown particles present are PBS salts. Azan Mallory staining. Magnification, 4×. When presented, the insets are at 10× magnification.

The LM analysis showed a uniform layer of healthy cells in all groups, with differences between them (Figure 2).

At 24 h of culture, the control group displayed densely packed cells, with a fusiform morphology and small dimensions. Instead, all experimental groups showed slightly larger cells distant from each other. In the CGF and APG groups, sporadic polygonal cells with cytoplasmatic extensions were particularly evident.

At 72 h, the control and L-PRF cells overlapped with the homologous 24 h ones, while CGF and APG appeared to result in more polygonal cells and a composite network of cytoplasmic processes.

At 7 days, the culture of the control group showed fusiform cells, while almost all the fibroblasts cultured in the CGF and L-PRF groups were large and polygonal. The APG group also displayed few fusiform cells by this point.

### 3.3. Morphological Analysis—SEM

**Figure 3:**
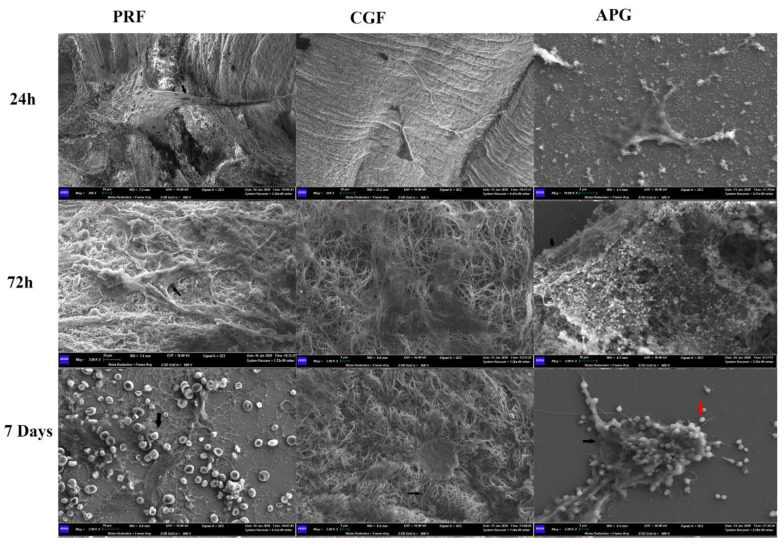
APC representative SEM images showing the morphological reaction of the fibroblasts to the fibrin network, assuming a polygonal shape. Morphological differences of the fibrin networks of the three APCs are evident, with the presence of blood cells with PRF and of platelets with APG. The black arrows indicate the cellular projections toward the biomaterials. The red arrow indicates the platelets in the APG. It is interesting to highlight, in the APG samples, the morphological reaction of the fibroblasts trying to envelop the platelets.

Regardless of the APC used, the SEM analysis revealed that all the experimental groups showed abundant, flattened and healthy cells (Figure 3).

After 24 h of culture, both polygonal and elongated cells were detectable. All cells displayed thick cytoplasmic digitations, attributable to developing lamellipodia.

In the 72 h groups, the cells were larger and anchored to APCs by means of numerous cell contacts. The latter included both thin cytoplasmic digitations, such as filopodia, and larger digitations, such as lamellipodia.

After 7 days of culture, the cells exhibited an extensive network of intercellular contacts. Filopodia and lamellipodia were more abundant, and higher magnification micrographs revealed the intimate relationship between cell digitations and the elements of the APCs.

### 3.4. Morphological Analysis—CLSM

**Figure 4:**
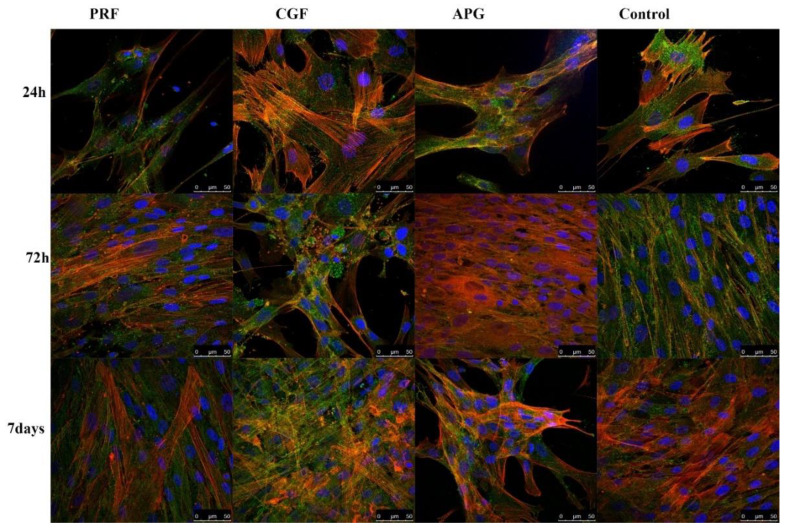
Confocal laser scanning microscopy (CLSM) images of actin filaments revealed by phalloidin in control and treated HPLF at different times, including the HPLF under the three considered conditions and the controls at the different times. Low magnification. CD90, used as an HPLF marker, is green stained. The actin is intensively represented, compared to the control. It is interesting to note how the fibroblasts in contact with the PRF show an intense stain of the actin inside the cellular projections, already at 72 h and more so at 7 days; the actin of the cells exposed to CGF is instead heavily modified and solicited; the APG has a strong representation of the actin fibers at 72 h.

The CLSM analyses highlighted both the status of the nuclei (blue) and the distribution of the actin (red). The nuclei appeared oval or rounded (blue) and well represented in all samples, while actin staining was different between the groups. The control group displayed a weak red signal; only after 7 days of culture was a slight increase was noticeable. In all APC samples in general, a progressive intensification of the red signal occurred during the culture period (Figure 4).

**Figure 5:**
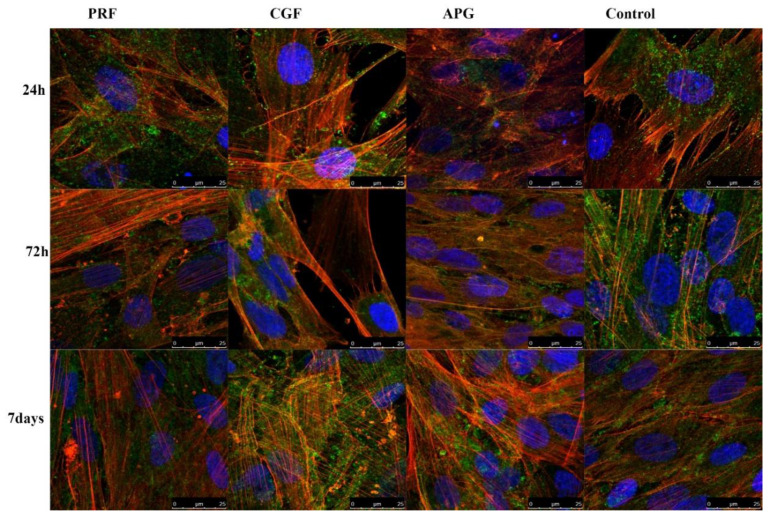
CLSM representative pictures, at a higher magnification, of actin filaments in treated HPLF and control. It is worth noting how in the CGF 72 h observations, the spaces between the cells are filled with large cytoplasmic extensions, displaying a delicate and uniform red signal.

Higher magnifications (Figure 5) allowed the evaluation of the detailed intracellular and intercellular distribution of actin. In the control group, it was detected mainly in short projections that remained close to the cellular body (*green—CD90*) and beneath the plasma membrane. At Day 7, despite an increase in the red signal, the cells appeared densely packed and no overlapping of projections were visible. In APCs, the samples’ red signals were strongly present in cytoplasmic projections, forming bundles arranged parallel to the major axis of the cellular extensions. The projections passed on neighbouring cells and overlapped each other, forming a rich three-dimensional network between different cells. In the CGF and PRF samples (especially after 72 h and 7 days), the nuclei were distant from each other and the space between the cell bodies, besides the definite cellular protrusions, was filled with large cytoplasmic extensions, displaying a delicate and uniform red signal. The APG nuclei were closer to each other, with a distribution almost similar to the control ones.

## 4. Discussion

APCs represent an autologous biomaterial with a high potential for regeneration. The APCs indeed contain a polymer of fibrin matrix, with a high concentration of growth factors [5,14,19,33].

The use of APCs is frequent in therapies and interventions with regenerative aims [34]. However, the efficacy of these protocols represents the core of the scientific debate. The APG, the PRF and the CGF similarly release growth factors including the platelet-derived growth factor (PDGF), transforming growth factor β (TDF-β), insulin-like growth factor-1 (ILGF-1), vascular endothelial growth factor (VEGF), and epidermal-growth factor (EGF), which show chemotactic activity and influence cellular differentiation and proliferation [10].

A key factor influencing the different regenerative potential of the APCs is the release time for the growth factors; as previously reported in the literature, the APG indeed releases the growth factors quickly, but the PRF and CGF release the growth factors slowly [10]. This difference in timing is due to the different time taken for fibrin polymerisation.

As reported by Kobayashi et al., the APG types of APC release a great amount of growth factors in the first hour, with respect to PRF [10].

The regenerative induction is therefore dissimilar, requiring the use of different materials according to the specific clinical situation. Indeed, according to the pre-determined treatment plan, the timing of the growth factor release determines the choice of the material to use regarding performance; if the clinical situation requires fast regeneration but no further intervention later in time, APG might be a choice; instead, if the clinical situation requires several surgical interventions during the time, PRF or CGF may represent a better choice.

This is reflected by the results from our in vitro study.

Indeed, the XTT assay showed a difference in the proliferation of HPLF among three tested APCs at different times.

Interestingly, the XTT assays showed an intense proliferation of PRF within 24 h, which subsequently decreased. These data could be due to the high spin protocol, which can reduce the number of platelets at the expense of a very thick fibrin matrix, mostly used as a membrane or graft in socket preservation or in treating periodontal defects [6,20]. For example, as reported by Sharma et al., the use of PRF associated with an open flap technique showed a statistically significant result with a greater reduction of the periodontal depth than at control sites (open flap alone), with a difference of 2.17 mm [24].

The CGF fibrin matrix, instead, presents a lower density compared to the PRF one; hence, the fibrin network is not able to hold a higher volume of platelets and growth factors [35].

From the studies in literature, the APG showed an early release of growth factors, and this could be associated with the low density of the fibrin matrix in which the platelets are not trapped and can easily release growth factors without any obstacles [36]. The advantage of using APG is in the activation of biomaterials such as bovine bone grafts through the formation of a gel incorporating bone chips, facilitating insertion during interventions for ridge preservation, sinus floor augmentation or treating mandibular II degree furcation [37]. As reported by Pradeep et al., the use of APG combined with bovine bone grafts showed a lack of complete closure of the furcation defects, implying a limited role of autologous APG as a regenerative material in the periodontal field [17].

The observations from the LM and SEM images showed important morphological changes related to the presence of APCs, in addition to the proliferation data provided by the XTT assay. In all experimental settings, the observed HPLF spreading suggests the cells’ ability to move over the surface of APCs. The differences observed in the spreading time could be ascribed to the surrounding microenvironment [38]. The latter depends on the physical properties of the APCs, e.g., the stiffness and thickness, which differ significantly between substrates [39].

Moreover, the increase in cell body dimensions, the shifting into a polygonal shape, and the development of several cellular projections are signs of cellular activation and interaction with the extracellular environment. The above findings reveal a fine balance between motility and cell adaptive adhesion to the APC components.

The CLSM images strongly support and clarify the LM and SEM observations. The progressive increase in and the dynamic distribution of actin prove the cytoskeletal remodelling sustained by APCs.

As shown by the controls at 24 h, 72 h and 7 days, the expression of the actin appears to be physiological and with a stable morphology.

The observations of the cells in culture with the APCs showed not only an intense expression of actin at all the considered times but also a re-organisation of the intracellular actin protein that was displayed inside the projections. These data, together with the observation of the nuclei, support the induction of proliferation by APCs and confirm the modification of the cytoskeleton already observed in the SEM images.

Indeed, the actin, generally, represents the protein of the cytoskeleton responsible for the modification of the morphology of the cells and for the migration and the movement of cell organelles and is considered a conclusive indicator of fibroblast activation [40,41,42].

The development of cellular projections, especially lamellipodia, is determined by cytosolic actin.

In the wound, the involved cytokines have key chemotactic and repairing roles for the subsequent regenerative processes. In particular, growth factors such as PDGF and TGF-β, released during wound healing, facilitate cellular proliferation and migration and collagen synthesis in fibroblasts [43,44].

Our findings revealed the interaction between the chemical-physical properties of three different APCs and relative fibroblast activation. All the examined materials appeared suitable for tissue regeneration techniques. Moreover, the differences observed in terms of proliferation, spreading and fibroblast activation may inform the choice of the appropriate APC according to specific clinical needs in regenerative periodontal interventions.

## 5. Conclusions

Considering the limitations of this study, the results obtained indicate that all the APCs tested seem to be suitable as scaffold materials for fibroblast cell culture. These findings suggest that APCs may be reliable materials for use in guided tissue regeneration techniques in periodontology. Further studies, randomized clinical trials, are needed to confirm these results.

## Figures and Tables

**Table 1:** Resuming table of the properties of each APCs.

Blood Product	L-PRF	CGF	APG
Protocol	2700 rpm for 12 min	2400 rpm alternated	2400 rpm
		to 3000 rpm for 14 min	10 min
Flow	One step continuous	One step discontinuous	Two step clot activation with CaCl_2_
PDGF levels [27] (ng/mL)	High	Highest	Low
VEGF levels [27] (pg/mL)	Highest	High	Low
TGF-β 1 [27] (ng/mL)	High	Highest	Low
Speed-rate	Fast	Mixed	Fast
Reproducibility	No bias	No bias	Possible bias
Use of Anticoagulants	NO	NO	YES
Fibrin density	High	Medium	Low
Speed of fibrin formation	High	High	Low
Fibrin morphology	Tetramolecular	Tetramolecular	Tetramolecular
Dose	9 mL	9 mL	9 mL
Handling	Easy	Easy	Complex
Expense	Low cost	Low cost	High cost
Young’s modulus [27]	0.35 Gpa	70 kpa	60 kpa

PDGF: platelet-derived growth factor, VEGF: vascular endothelial growth factor, TGF-β 1: transforming growth factor β 1.

## Supplementary Materials

The following are available online at https://www.mdpi.com/1996-1944/13/10/2282/s1, Table S1: The parameter “biomaterials” which compares the proliferation values of the cells cultured with the examined APCs vs the control showed a *p* value <0.05, Table S2. Dunnett’s multiple comparison test showed how the means of proliferation of CGF vs CONTROL was statistically significant at 7 days.
