# Supplementary material for: Evaluation of Different Autologous Platelet Concentrate Biomaterials: Morphological and Biological Comparisons and Considerations
Source: Materials (Basel). 2020 May 15;13(10):2282. doi: 10.3390/ma13102282 (PMC7288147; doi:10.3390/ma13102282)
Supplement: Supplementary file 1 [file materials-13-02282-s001.pdf]

Table S1. The parameter “biomaterials” which compares the proliferation values of the cells cultured with the examined APCs vs the control showed a p value <0.05.

ANOVA Table	SS	DF	MS	F (DFn, DFd)	P Value
Times x BIOMATERIAL	0.17	6	0.02	F (6, 16) = 0.75	P=0.61
Times	0.15	2	0.07	F (2, 16) = 1.99	P=0.16
BIOMATERIAL	1.09	3	0.36	F (3, 8) = 4.18	P=0.04

Table S2. Dunnett’s multiple comparison test showed how the means of proliferation of CGF vs CONTROL was statistically significant at 7 days.

Dunnett's Multiple Comparisons Test	Mean Diff.	95.00% CI of Diff.	Significant?	Summary	Adjusted P Value
24h					
CONTROL vs. PRF	-0.02	-0.50 to 0.45	No	ns	0.99
CONTROL vs. CGF	-0.23	-0.71 to 0.24	No	ns	0.48
CONTROL vs. APG	0.12	-0.35 to 0.60	No	ns	0.84
72h					
CONTROL vs. PRF	-0.20	-0.68 to 0.27	No	ns	0.57
CONTROL vs. CGF	-0.42	-0.89 to 0.05	No	ns	0.09
CONTROL vs. APG	-0.04	-0.52 to 0.43	No	ns	0.99
7days					
CONTROL vs. PRF	0.039	-0.43 to 0.51	No	ns	0.99
CONTROL vs. CGF	-0.53	-1.01 to -0.05	Yes	*	0.02
CONTROL vs. APG	0.07	-0.40 to 0.55	No	ns	0.95
